# Enhancing hematoma expansion prediction in hypertensive intracerebral hemorrhage based on habitat and perihematomal edema radiomics from non-contrast CT: a dual-center study

**DOI:** 10.3389/fnins.2026.1825573

**Published:** 2026-06-24

**Authors:** Yangyingqiu Liu, Jinfeng Cao, Tao Feng, Yu Bing, Yuxuan Li, Qun Shang, Jiaqi Li, Peng Sun, Donghao Song, Yu Wang, Yanwei Miao, Xin Luo

**Affiliations:** 1 Department of Radiology, Zibo Central Hospital, Zibo, China; 2 Department of Thoracic Surgery, Zibo Central Hospital, Zibo, China; 3 Department of Radiology, Central Hospital of Dalian University of Technology, Dalian, China; 4 School of Medical Imaging, Shandong Second Medical University, Weifang, China; 5 School of Medical Imaging, Binzhou Medical University, Binzhou, China; 6 Department of Neurosurgery, Zibo Central Hospital, Zibo, China; 7 Siemens Healthineers, Shanghai, China; 8 Department of Radiology, First Affiliated Hospital of Dalian Medical University, Dalian, China

**Keywords:** habitat imaging, hematoma expansion, hypertensive intracerebral hemorrhage, perihematomal edema, radiomics

## Abstract

**Objectives:**

Characterizing the microenvironmental habitats within the hematoma may yield crucial imaging biomarkers and improve the early prediction of hematoma expansion (HE) in patients with hypertensive intracerebral hemorrhage (HICH). Our objective was to construct and validate a combined model that integrates clinical data with whole-hematoma radiomics, habitat radiomics of the hematoma, and perihematomal edema (PHE) radiomics features extracted from non-contrast computed tomography (NCCT) images for preoperative HE prediction.

**Methods:**

This retrospective dual-center cohort of 353 HICH patients. Based on baseline NCCT images, radiomics features were extracted from the whole hematoma, three distinct habitats within the hematoma, and the PHE region. Five models were constructed: a clinical model, a whole-hematoma radiomics model, a habitat-based radiomics model, a PHE radiomics model, and a combined model. Model performance was evaluated using receiver operating characteristic (ROC) curve analysis.

**Results:**

The combined model integrated with smoking history, island sign, maximum distance of the PHE, and the whole-hematoma, habitat, and PHE radiomics models, achieved the best predictive performance. In the training, testing, and validation sets, the combined model predicted the area under the curve for HE as 0.951 (95% CI: 0.915–0.986), 0.937 (95% CI: 0.883–0.991), and 0.939 (95% CI: 0.888–0.989), respectively.

**Conclusion:**

The NCCT-based combined model integrating clinical data, whole-hematoma radiomics, habitat radiomics, and PHE radiomics improves HE prediction in patients with HICH, providing a noninvasive tool with potential for guiding treatment strategies.

## 1 Introduction

Spontaneous intracerebral hemorrhage (SICH) is a seriousneurological disorder liable for approximately 10 to 15% of all stroke incidents (Kubiszewski et al., 2020). Hypertensive intracerebral hemorrhage (HICH) constitutes the SICH predominant etiology, accounting for 50–70% of instances. Patients with SICH may encounter elevated rates of disability and fatality (Kase and Hanley, 2021; Kittner et al., 2021). Hematoma expansion (HE) plays a crucial role in the deterioration of ICH patients and is related to worse outcomes, occurring in 13–38% of cases (Li et al., 2020). HE represents a prospective treatment target in clinical trials (Wang et al., 2021). Therefore, precise prediction of HE events can be a key basis for formulating therapeutic approaches (Feng et al., 2023).

Neuroimaging is critical for diagnosing and determining the underlying cause (Thabarsa et al., 2023). Owing to its rapid speed, extensive availability, and superior sensitivity and specificity in identifying acute blood (de Oliveira Manoel et al., 2016), non-contrast computed tomography (NCCT) represented the preferred methodology for assessing changes in cerebral hematomas (Zhang et al., 2018). Presently, the early prediction of HE is frequently conducted using traditional CT image signs (Wei et al., 2020; Zhang et al., 2020; Shakya et al., 2021; Yang et al., 2020). Nonetheless, these signs are semi-quantitative and subjective in nature, with limited sensitivity and specificity for early HE prediction, and poor early stratification. These inherent limitations restrict their reliability for informed clinical decision-making.

Advancements in radiomics and their implementation in clinical research recently have introduced innovative methodologies for the quantitative measurement and prediction of HE (Feng et al., 2023; Yu et al., 2023; Ye et al., 2023). Prior research has shown that the radiomics model surpassed the majority of NCCT signs in forecasting HE (Jiang et al., 2022; Liu et al., 2023). Traditional radiomics methodologies often emphasize comprehensive lesion analysis, but they often neglect sub-regional phenotypic changes, thus constraining their therapeutic utility. A novel approach, in contrast to previous methods, segments tissues into subregions termed habitats, which are delineated by quantitative imaging markers to represent distinct spatial areas exhibiting analogous biological characteristics within the lesion, thereby facilitating the visualization and quantification of intralesional heterogeneity (Zhang et al., 2023; Gao et al., 2023). Recent studies indicate that habitat imaging holds significant promise in forecasting tumor metastasis and recurrence, alterations in the microenvironment, and responses to treatment, particularly in central nervous system tumors, as well as breast and liver cancers (Lee et al., 2023; Cho et al., 2022; Zhang et al., 2023). Recent studies indicate that habitat imaging holds significant promise in forecasting tumor metastasis and recurrence, alterations in the microenvironment, and responses to treatment, particularly in central nervous system tumors, as well as breast and liver cancers. Beyond oncology, habitat radiomics has shown emerging value in neuroimaging, including identifying high-risk vulnerable intracranial atherosclerotic plaques for stroke risk prediction (Gao et al., 2023), stratifying symptomatic re-hemorrhage risk in brainstem cavernous malformations (Gui et al., 2026). Perihematomal edema (PHE) appears as a hypodensity rim surrounding the hematoma on NCCT, reflecting increased water content in the brain tissue adjacent to the hemorrhage. PHE is recognized as a quantifiable biomarker of brain injury and a potential therapeutic target (Cordonnier et al., 2018; Huang et al., 2023). Notably, microstructural alterations within the PHE region have been demonstrated to be significantly associated with HE (Hervella et al., 2020; Morotti et al., 2019; Wan et al., 2022). However, to the best of our knowledge, no prior study has integrated clinical information, whole hematoma radiomics features, habitat radiomics features, and PHE radiomics features for the prediction of HE.

Therefore, we hypothesized that characterizing the microenvironmental habitats within both the hematoma and the PHE could yield valuable predictive markers and improve the prediction of HE in patients with HICH. Accordingly, the present study aimed to develop a combined model that integrates clinical data with whole hematoma radiomics, habitat radiomics of the hematoma, and PHE radiomics features extracted from NCCT images for the preoperative prediction of HE.

## 2 Methods

This retrospective dual-center study was approved by the institutional review boards of both participating centers, and the requirement for informed consent was waived.

### 2.1 Patients

This investigation retrospectively included 226 HICH cases diagnosed and treated at Zibo Central Hospital (Center A) between January 2021 and December 2023, categorized randomly (7:3) into training and testing sets. Additionally, 127 cases of HICH treated at the First Affiliated Hospital of Dalian Medical University (Center B) from January 2018 to December 2019 were retrospectively collected for the validation set.

Inclusion criteria: (1) A history of hypertension, characterized by a systolic pressure of 140 mmHg or above, or diastolic pressure of 90 mmHg or above; (2) Baseline CT scan conducted within 6 h of disease onset; (3) Within 24 h of the baseline CT, a re-examination was performed; (4) Hemorrhage located in the deep brain parenchyma, including the basal ganglia, thalamus, internal capsule, and deep periventricular white matter; (5) Complete clinical data.

Exclusion criteria were as follows: (1) Hemorrhage due to tumors, aneurysms, cerebrovascular malformations, trauma, and ischemic cerebral infarction with hemorrhagic transformation; (2) Surgical treatment prior to the CT examination or re-examination; (3) Poor image quality, defined as motion artifacts causing substantial blurring that compromised delineation of the hematoma boundary and/or the perihematomal region, severe streak artifacts obscuring the hemorrhage or perihematomal area, incomplete NCCT scans, or other substantial artifacts that degraded the diagnostic interpretability of the images; and (4) Hematoma with poorly demarcated boundaries that precluded reliable segmentation.

Hematoma volume was measured at the initial diagnosis for all patients who were newly diagnosed with HICH, and measured again during a follow-up CT scan conducted within 24 h. An early HE was identified by a hematoma volume growth of either ≥ 33% or ≥ 6 mL within 24 h follow-up CT compared to baseline (Morotti et al., 2021). Patients meeting these criteria were classified into the HE group, while those who did not were categorized into the non-hematoma expansion (NHE) group.

### 2.2 Equipment

Image collection was performed using four CT scanners. Specifically, at the Center A, 191 cases were scanned using the Ingenuity 64-slice spiral CT (Philips Healthcare, Netherlands), and 35 cases were scanned by the Incisive 64-slice spiral CT (Philips Healthcare, Netherlands). At the Center B, we scanned 86 cases using the Somatom Emotion 16-slice spiral CT (Siemens Healthcare, Germany), and 41 cases underwent the Lightspeed VCT 64-slice spiral CT (GE Healthcare, USA). The scan encompassed the region from the base of the skull to the vertex of the head, using a slice thickness of 1 mm with no interslice gap, field of view of 250 mm × 250 mm and an acquisition matrix of 512 × 512. The tube voltage was set at 120 kV, and the tube current varied between 200 and 230 mAs.

### 2.3 Clinical model construction

#### 2.3.1 Clinical data and conventional imaging features

Clinical data were retrospectively gathered from the hospital’s electronic medical records system, covering age, gender, systolic and diastolic blood pressure, smoking history, onset to baseline NCCT time. All information was provided at admission by a neurologist trained in stroke diagnosis and management.

Conventional imaging features were assessed by two radiologists with 10 and 20 years of experience in neuroimaging diagnosis (radiologist 1 and radiologist 2, respectively). On baseline NCCT images, the conventional imaging features including island sign, black hole sign, blend sign were recorded (Huang et al., 2023), and the maximum distance of the PHE (from the outer margin of the hematoma to the outer edge of edema on axial baseline NCCT images) (Dahlberg et al., 2022), baseline hematoma volume (Webb et al., 2015) were measured.

#### 2.3.2 Clinical features and clinical model construction

First, the interobserver agreement of the conventional imaging features evaluated by radiologist 1 and radiologist 2 was assessed using the intraclass correlation coefficient (ICC). Feature with an ICC greater than 0.75 were retained for subsequent analysis. Clinical and conventional imaging features with *p* < 0.05 in univariate analysis were included in multivariate logistic regression through the backward stepwise elimination method. The clinical model was established by applying multivariate logistic regression.

### 2.4 Whole hematoma segmentation and radiomics feature extracted

The images were resampled to a resolution voxels of 1 × 1 × 1 mm^3^ using bi- cubic B-spline interpolation to standardize the spatial resolution for subsequent analysis. The regions of interest (ROI) were delineated using ITK-SNAP version 3.8.0.^1^ The process of image segmentation was carried out manually, tracing the ROI along the hematoma boundary on each axial CT slice. Radiologist 1 delineated the ROI independently, blinded to all clinical data and outcome labels. A total of 107 radiomic features were extracted from each ROI (see Supplementary material). The Image Biomarker Standardization Initiative (IBSI) guidelines were followed while extracting features in image processing (Zwanenburg et al., 2020).

An initial consistency test was carried out on the radiomics features of the whole hematoma to ensure stability in measurements among observers. The consistency of these features was assessed through the ICC. For inter-observer agreement, radiologist 3 (with 15 years of experience in neuroimaging diagnosis) independently segmented the ROI in all patients, blinded to all clinical data and outcome labels, and feature consistency was assessed using ICC. For further analysis, those features with an ICC greater than 0.75 were preserved (Wittenbecher et al., 2022), while others were excluded.

### 2.5 Habitat clustering and raidomics feature extracted

Habitat clustering and radiomic feature extraction were carried out using MPQuan-Habitat software (Chengdu ZhongYing Medical Technology Co., Ltd., Chengdu, China). The most suitable number of clusters was identified at the group-level using the Silhouette score algorithm. Patient-specific habitat maps were generated through group-level clustering. Voxel-wise clustering was performed using the K-means algorithm with a predefined cluster count of three, yielding three distinct habitat ROIs per patient. Radiomic features were obtained from each individual habitat subregion. A total of 321 radiomic features were extracted, with 107 features were extracted from each ROI. The IBSI guidelines were followed while extracting features (Zwanenburg et al., 2020).

### 2.6 PHE ROI dilation and radiomic feature extraction

PHE ROI delineation and radiomic feature extraction were performed using MPQuan-Habitat software (Chengdu ZhongYing Medical Technology Co., Ltd., Chengdu, China). Starting from the boundary of the manually segmented hematoma ROI, an automated isotropic three-dimensional dilation algorithm was applied to generate three concentric PHE ROIs with thicknesses of 5 mm, 10 mm, and 15 mm, respectively. From each of these PHE ROIs, 107 radiomic features were extracted. The IBSI guidelines were followed while extracting features (Zwanenburg et al., 2020).

### 2.7 Radiomics feature selection and model development

Radiomics feature selection was performed separately for the whole hematoma, habitat subregions, and the three PHE ROIs (PHE 5 mm, PHE 10 mm, and PHE 15 mm). For each ROI, dimensionality reduction was conducted through the following sequential steps. First, to eliminate redundancy, a Pearson correlation matrix was computed for features. For any feature pair with an absolute correlation coefficient |*r*| > 0.9, one feature was retained and the other removed. Then, the retained features were subsequently subjected to the least absolute shrinkage and selection operator (LASSO) regression with 5-fold cross-validation, which identified the most informative features by shrinking the coefficients of non-predictive features to zero. Based on the LASSO results, features with non-zero coefficients were selected to construct five radiomic scores (rad-scores) by summing each selected feature value multiplied by its corresponding coefficient: whole hematoma ROI radscore (whole_radscore), habitat subregions ROI radscore (habitat_radscore), PHE 5 mm ROI radscore (PHE 5mm_radscore), PHE 10 mm ROI radscore (PHE 10mm_radscore), PHE 15 mm ROI radscore (PHE 15mm_radscore). Among the three PHE ROIs based rad-scores, the one demonstrating the best predictive performance was selected and designated as PHE_radscore. Subsequently, the whole hematoma radiomics model was built using whole_radscore, the habitat radiomics model using habitat_radscore, and the PHE radiomics model using the best predictive performance PHE_radscore.

A combined model was then constructed via logistic regression by integrating the whole-hematoma radiomics model, the habitat radiomics model, the PHE radiomics model, and the clinical model. To enhance clinical applicability, the combined model was visualized as a nomogram.

### 2.8 Model evaluation

The discriminatory performance of the clinical model, whole hematoma radiomics model, habitat radiomics model, PHE radiomics model and combined model was analyzed using the receiver operating characteristic (ROC) curve analysis. For each model, the optimal probability threshold for binary classification was determined using the Youden index. Key performance metrics—including area under the curve (AUC), sensitivity, specificity, accuracy, positive predictive value, and negative predictive value—were computed. Nagelkerke *R*^2^ was used to assess the relative *R*^2^ contributions to variance from clinical model, whole hematoma radiomics model, habitat radiomics model, PHE radiomics model in the combined model. The calibration of the combined model was assessed with calibration curves, Hosmer–Lemeshow test and the Brier score. The combined model’s clinical utility was further ascertained via decision curve analysis (DCA). The DeLong test served to evaluate AUC differences between the models.

**Figure 1:**
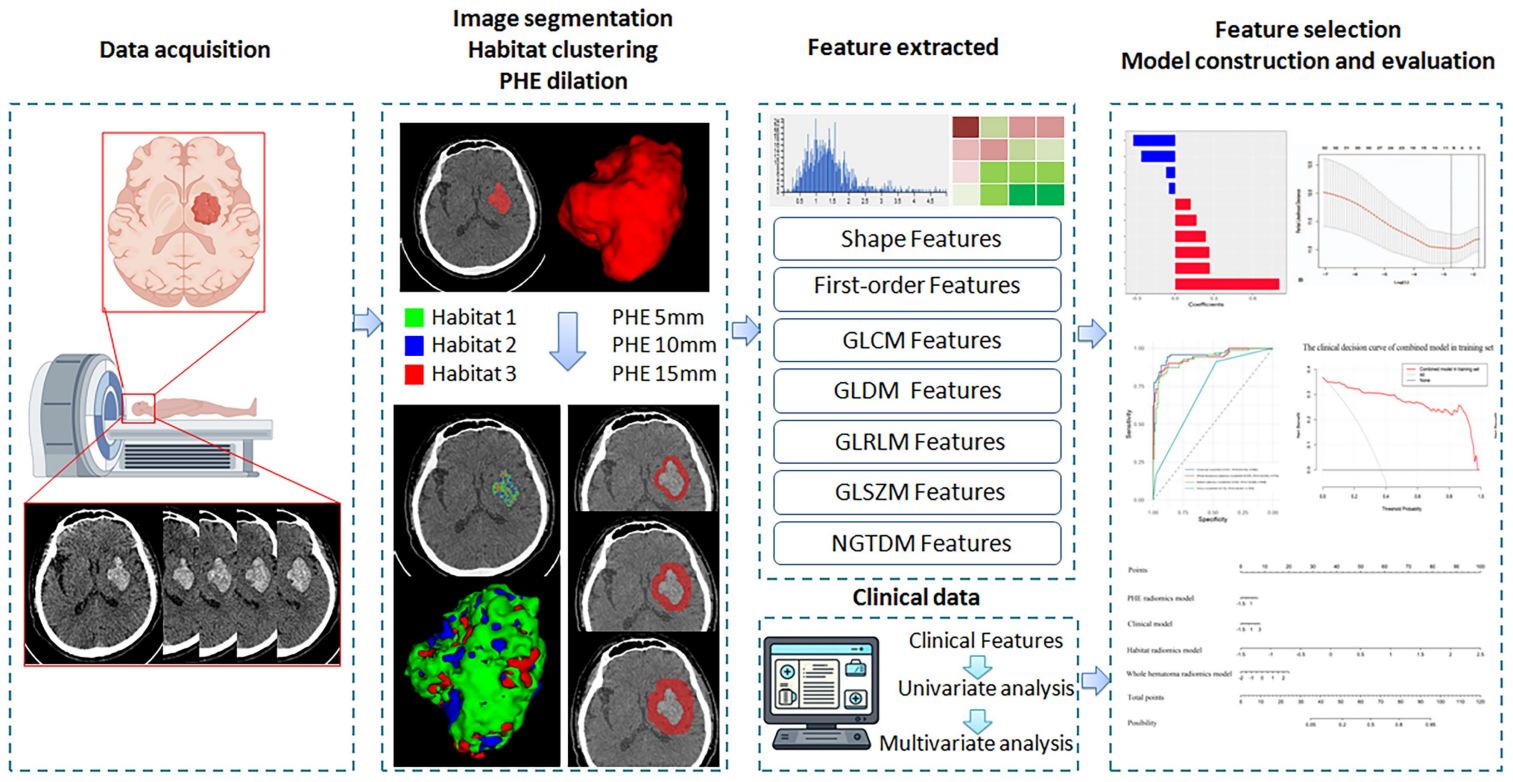
Study flow chart. ICH, intracerebral hemorrhage; PHE, perihematomal edema; GLCM, gray level co-occurrence matrix; GLDM, gray level dependence matrix; GLRLM, gray level run length matrix; GLSZM, gray level size zone matrix; NGTDM, neighboring gray tone difference matrix.

Figure 1 depicts the overall workflow of the study. Following manual lesion segmentation, the entire subsequent procedure was fully automated to ensure reproducibility and consistency across operators.

### 2.9 Statistical analysis

Statistical analyses were conducted using R version 4.0.0.^2^ For comparisons between two groups, the independent samples t-test was used for normally distributed continuous variables, and the Mann–Whitney U test was applied for non-normally distributed continuous variables. The chi-square test was employed for categorical variables. For comparisons among the three sets, normally distributed continuous variables with homogeneity of variance were compared using one-way analysis of variance (ANOVA). Non-normally distributed continuous variables were compared using the Kruskal–Wallis H test. Categorical variables among the three sets were compared using the chi-square test. A two-sided *p* < 0.05 was considered statistically significant.

## 3 Results

### 3.1 Patient characteristics

A total of 353 patients were included in this study, with 158 patients assigned to the training set, 68 to the testing set, and 127 to the validation set. The demographic and clinical characteristics of the patients are shown in Table 1.

**Table 1:** Patient demographic and clinical characteristics.

Characteristics	Training set				Testing set				Validation set				*F*/*X*^2^	*p*
	HE (*n* = 54)	NHE (*n* = 104)	*t*/*z*/*x*^2^	*p*	HE (*n* = 23)	NHE (*n* = 45)	*t*/*z*/*x*^2^	*p*	HE (*n* = 43)	NHE (*n* = 84)	*t*/*z*/*x*^2^	*p*		
Age (years)	60.98 ± 14.45	62.27 ± 13.39	0.558	0.578	58.52 ± 16.94	59.22 ± 13.66	0.184	0.854	56.30 ± 14.81	60.25 ± 14.30	1.455	0.148	1.805	0.166
Gender			0.154	0.695			0.808	0.369			0.398	0.528	0.386	0.824
Male	38 (70.37%)	70 (67.31%)			15 (65.22%)	34 (75.56%)			32 (74.42%)	58 (69.05%)				
Female	16 (29.63%)	34 (32.69%)			8 (34.78%)	11 (24.44%)			11 (25.58%)	26 (30.95%)				
Onset to CT time (h)	4.00 (1.50)	3.50 (1.50)	−0.331	0.741	3.50 (1.50)	4.00 (2.00)	−0.020	0.984	4.00 (1.00)	3.00 (1.00)	−1.504	0.133	5.782	0.056
Systolicpressure (mmHg)	157.00 (35.50)	155.50 (26.75)	−0.847	0.397	160.00 (46.00)	155.00 (24.50)	−0.538	0.590	157.00 (42.00)	158.00 (37.00)	−0.298	0.766	2.161	0.339
Diastolicpressure (mmHg)	90.00 (19.00)	89.50 (19.75)	−0.048	0.962	90.00 (24.00)	93.00 (19.50)	−0.506	0.613	93.00 (18.00)	91.00 (23.00)	−0.899	0.369	0.934	0.627
Smoking history [*n*(%)]	9 (16.67%)	5 (4.81%)	6.190	0.013	6 (26.09%)	3 (6.67%)	4.999	0.025	10 (23.26%)	7 (8.33%)	5.463	0.019	1.739	0.419
Island sign [*n*(%)]	19 (35.19%)	7 (6.73%)	20.933	<0.001	8 (30.43%)	4 (8.89%)	7.022	0.008	13 (30.23%)	10 (11.90%)	6.442	0.015	0.143	0.931
Black hole sign [*n*(%)]	27 (50.00%)	42 (40.38%)	1.336	0.310	16 (69.56%)	21 (46.67%)	3.217	0.073	16 (37.21%)	35 (41.67%)	0.235	0.628	3.719	0.156
Blend sign [*n*(%)]	13 (24.07%)	23 (22.12%)	0.078	0.842	6 (26.09%)	12 (26.67%)	0.003	0.959	5 (11.63%)	16 (19.05%)	1.134	0.287	3.017	0.221
Maximum distance of the PHE (mm)	10.50 (6.00)	7.00 (5.00)	−4.218	<0.001	10.00 (6.00)	8.00 (4.00)	−2.195	0.028	9.00 (6.00)	7.00 (3.00)	−2.770	0.006	4.778	0.092
Baseline hematoma volume (ml)	17.84 (20.45)	7.50 (10.57)	−4.626	<0.001	22.16 (25.03)	9.39 (12.06)	−4.712	<0.001	18.27 (17.93)	10.63 (16.61)	−3.255	0.001	2.903	0.234

HE, hematoma expansion; NHE, non-hematoma expansion; PHE, perihematomal edema.

### 3.2 Clinical model

Univariate/multivariate logistic regression analysis in the training set (Table 2) indicated that smoking history (*p* = 0.049), island sign (*p* = 0.014), and maximum distance of the PHE (*p* = 0.018) were independent clinical predictors of HE. Based on these variables, a clinical model was developed. The clinical model predicts an AUC of 0.761 (95% CI: 0.679–0.843) for predicting HE in the training, 0.680 (95% CI: 0.544–0.817) in testing, and 0.660 (95% CI: 0.555–0.764) in validation sets (Table 3).

**Table 2:** Univariate and multivariate LR Analysis of clinical characteristics in the training set.

Characteristics	Univariate LR			Multivariate LR		
	OR	95% CI	*p*	OR	95% CI	*p*
Sex	0.867	0.425–1.769	0.695			
Age	0.993	0.970–1.017	0.575			
Onset to CT time	1.033	0.709–1.503	0.867			
Systolic pressure	1.009	0.993–1.024	0.274			
Diastolic pressure	1.002	0.981–1.024	0.839			
Smoking history	3.96	1.256–12.489	0.019	4.006	1.004–15.974	0.049
Island sign	7.522	2.913–19.429	<0.001	4.081	1.325–12.572	0.014
Black hole sign	2.476	0.762–2.861	0.249			
Blend sign	1.117	0.513–2.428	0.781			
Maximum distance of the PHE	1.233	1.1116–1.363	<0.001	1.156	1.025–1.304	0.018
Baseline hematoma volume	2.439	1.638–3.632	<0.001	1.568	0.953–2.578	0.076

LR, logistic regression; OR, odds ratio; CI, confidence interval; PHE, perihematomal edema.

**Table 3:** Prediction performance of five models for early hematoma expansion in hypertensive intracerebral hemorrhage.

Cohort	Model	AUC (95% CI)	SEN	SPE	ACC	PPV	NPV
Training set	Clinical model	0.761 (0.679–0.843)	0.685	0.769	0.741	0.607	0.825
	Whole hematoma radiomics model	0.840 (0.779–0.902)	0.889	0.654	0.734	0.571	0.919
	PHE radiomics model	0.839 (0.775–0.904)	0.852	0.702	0.753	0.597	0.901
	Habitat radiomics model	0.950 (0.917–0.983)	0.907	0.885	0.892	0.803	0.949
	Combined model	0.951 (0.915–0.986)	0.926	0.894	0.905	0.820	0.959
Testing set	Clinical model	0.680 (0.544–0.817)	0.565	0.667	0.632	0.464	0.750
	Whole hematoma radiomics model	0.852 (0.763–0.941)	0.826	0.711	0.750	0.594	0.889
	PHE radiomics model	0.868 (0.772–0.963)	0.870	0.756	0.794	0.645	0.919
	Habitat radiomics model	0.930 (0.870–0.989)	0.783	0.889	0.853	0.783	0.889
	Combined model	0.937 (0.883–0.991)	0.826	0.844	0.838	0.731	0.905
Validation set	Clinical model	0.660 (0.555–0.764)	0.512	0.750	0.669	0.512	0.750
	Whole hematoma radiomics model	0.834 (0.758–0.910)	0.977	0.614	0.378	0.350	0.857
	PHE radiomics model	0.834 (0.759–0.909)	0.814	0.726	0.756	0.603	0.884
	Habitat radiomics model	0.911 (0.841–0.981)	0.791	0.964	0.906	0.919	0.900
	Combined model	0.939 (0.888–0.989)	0.837	0.929	0.898	0.857	0.918

AUC, area under the curve; SEN, sensitivity; SPE, specificity; ACC, accuracy; PPV, positive predictive value; NPV, negative predictive value; PHE, perihematomal edema.

### 3.3 Whole hematoma radiomics model

For the whole hematoma, 93 out of 107 radiomics features demonstrated high stability (ICC > 0.75) and were retained for further analysis. Forty-eight highly correlated features (Pearson correlation coefficient *r* > 0.9) were eliminated, leaving 47 features for selection. Using 5-fold cross-validated LASSO regression, 5 radiomics features were chosen. The Rad-score for the whole hematoma was computed by weighting these selected features with their respective coefficients to construct the whole hematoma radiomics model. This whole hematoma radiomics model achieved AUCs of 0.840 (95% CI: 0.779–0.902) in the training, 0.852 (95% CI: 0.763–0.941) in testing, and 0.834 (95% CI: 0.758–0.910) invalidation sets (Table 3).

### 3.4 Habitat radiomics model

For the hematoma habitat analysis, 321 radiomics features were extracted across the three habitats (107 per habitat). After removing 180 redundant features (*r* > 0.9), 141 features remained for selection. Through 5-fold cross-validated LASSO regression, 15 features were identified. A habitat-based Rad-score was developed to build the habitat radiomics model, which yielded AUCs of 0.950 (95% CI: 0.917–0.983) in the training, 0.930 (95% CI: 0.870–0.989) in testing, and 0.911 (95% CI: 0.841–0.981) in validation sets (Table 3).

### 3.5 PHE radiomics model

For the PHE 5 mm, 10 mm, and 15 mm analyses, after removing 60, 63, and 62 highly correlated features (*r* > 0.9), 47, 44, and 45 features remained for selection, respectively. Following 5 fold cross validated LASSO regression, 14, 20, and 15 features were chosen. To identify the best predictive performance radscore among the PHE 5mm_radscore, PHE 10 mm_radscore, and PHE 15 mm_radscore, we compared their performance in the training, test, and validation sets (Supplementary Table S1). The PHE 10 mm _radscore demonstrated the highest AUC of 0.839 (95% CI: 0.775–0.904) for predicting HE in the training, 0.868 (95% CI: 0.772–0.963) in testing, and 0.834 (95% CI: 0.759–0.909) in validation sets. Therefore, PHE 10 mm_radscore was selected to construct the PHE radiomics model, and was subsequently used to build the combined model.

### 3.6 Combined model

**Figure 2:**
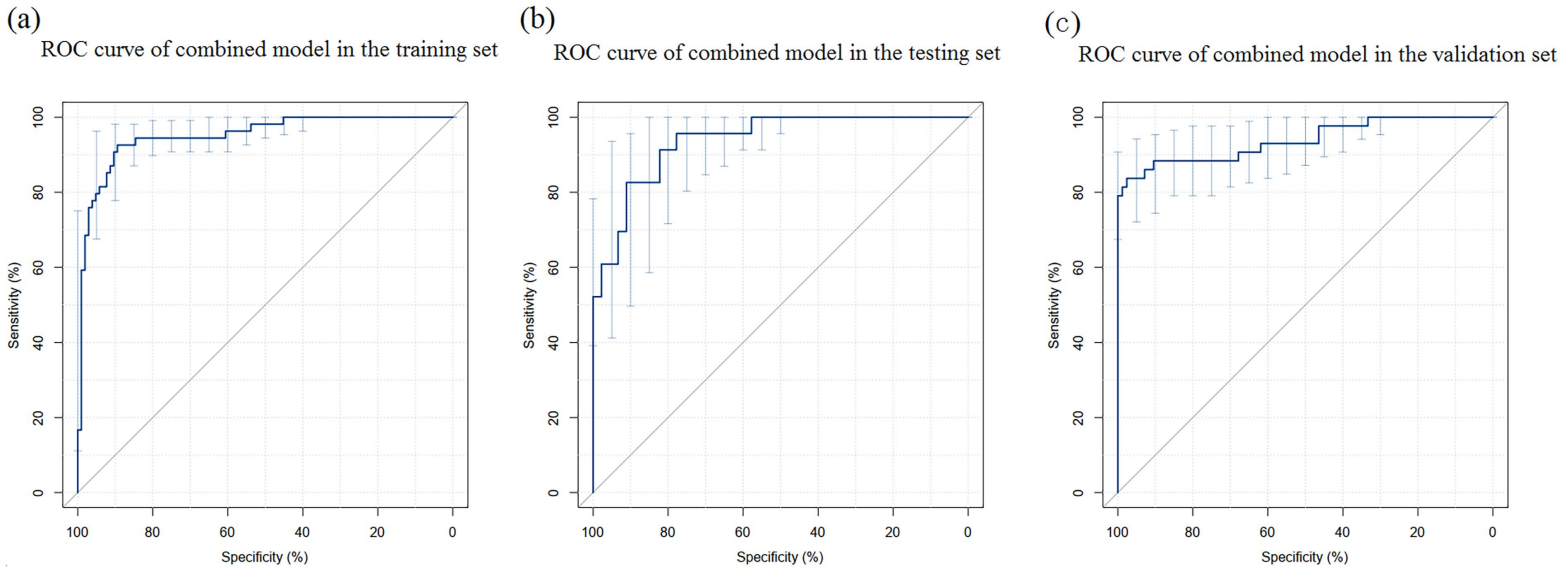
Receiver operating characteristic curve of the combined model in the training **(a)**, testing **(b)** and validation **(c)** sets.

The combined model was developed utilizing the clinical model, whole hematoma radiomics model, habitat radiomics model and PHE radiomics model. The combined model attained AUCs of 0.951 (95% CI: 0.915–0.986) in training, 0.937 (95% CI: 0.883–0.991) in the testing, and 0.939 (95% CI: 0.888–0.989) in validation sets (Figure 2). The confusion matrix of the combined model in the training, testing and validation set is presented in Supplementary Table S2.

The combined model demonstrated strong explanatory power in the training set, achieving a Nagelkerke *R*^2^ of 0.709. The dominance analysis revealed that clinical model, whole hematoma radiomics model, habitat radiomics model and PHE radiomics model contributed 12.55, 20.59, 47.39 and 19.47% of the total explained variance, respectively, with the habitat radiomics model contributing the largest proportion.

The calibration of the combined model was evaluated using calibration curves, the Hosmer–Lemeshow test, and the Brier score. The calibration curves demonstrated good agreement between the predicted probabilities and the observed outcomes in the training, testing, and validation sets (Supplementary Figure S1). The Hosmer–Lemeshow test yielded *p*-values above 0.05 in all three sets (*p* = 0.846, 0.183 and 0.356). The Brier scores were consistently below 0.10, with values of 0.074, 0.098, and 0.071 for the training, testing, and validation sets, respectively, further supporting the satisfactory overall calibration of the combined model.

**Figure 3:**
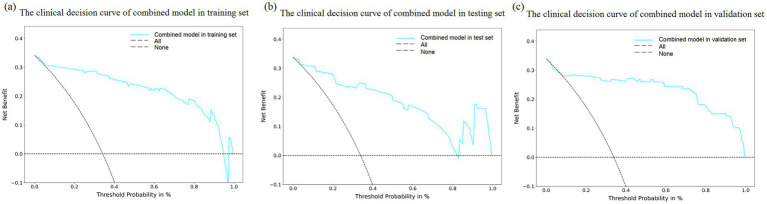
Decision curve analysis of the combined model across the training **(a)**, testing **(b)** and validation **(c)** sets.

**Figure 4:**
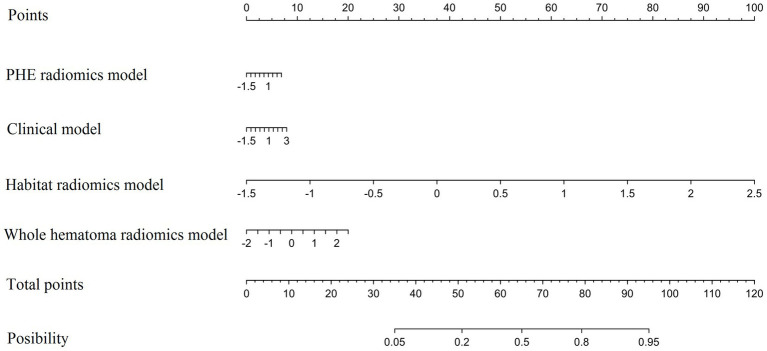
Nomogram of the combined model for hematoma expansion prediction.

DCA (Figure 3) revealed the most significant clinical net benefit for the integrated model across all datasets. A nomogram derived from the combined model (Figure 4) provides a valuable instrument for clinicians to assess the HE risk in HICH individuals.

### 3.7 Evaluation of the model’s prediction effectiveness

Table 3 summarizes the AUC (95% CI), sensitivity, specificity, accuracy, positive predictive value, and negative predictive value for all five models across the three datasets. The combined model consistently shows superior performance across all datasets.

The DeLong test findings indicated no significant variations in AUC among the training, testing, and validation sets (*p* = 0.687, *p* = 0.702 and *p* = 0.971, respectively) within the combined model. In the validation set, the combined model achieved a significantly higher AUC compared with the clinical model (ΔAUC = 0.279, 95% CI: 0.138–0.420, *p* < 0.001), the whole hematoma radiomics model (ΔAUC = 0.105, 95% CI: 0.043–0.167, *p* = 0.001), the habitat radiomics model (ΔAUC = 0.028, 95% CI: 0.001–0.055, *p* = 0.039), and the PHE radiomics model (ΔAUC = 0.105, 95% CI: 0.008–0.202, *p* = 0.035). The habitat radiomics model showed a numerically higher AUC than the whole hematoma radiomics model (ΔAUC = 0.077, 95% CI: −0.001 to 0.155, *p* = 0.054) and the PHE radiomics model (ΔAUC = 0.077, 95% CI: −0.035 to 0.189, *p* = 0.177), but these differences were not statistically significant.

## 4 Discussion

This study developed a composite model utilizing clinical data, conventional imaging features, whole hematoma radiomics features, PHE radiomics features and habitat radiomics features to forecast the probability of hemorrhagic expansion in individuals with HICH. All radiomics models showed substantial benefits above the clinical model. The combined model achieved an AUC of 0.939 (95% CI: 0.888–0.989) in the external validation cohort, demonstrating superior predictive performance for HE. The model is presented as a nomogram, serves as a trustworthy tool for clinicians to assess HE risk in HICH patients.

Previous studies (Yu et al., 2023; Wei et al., 2021) have discovered that patients who smoke have an elevated HE risk, which agrees with the outcomes of our investigation. Smoking may contribute to HE through various mechanisms: (1) nicotine-induced transient hypertension, (2) structural damage to vascular walls, (3) elevated blood–brain barrier (BBB) permeability due to chronic tobacco exposure, (4) nitric oxide in cigarette smoke further augmenting BBB permeability, and (5) exacerbation of secondary neurovascular injury following ICH. In addition, our study found that the island sign was an independent predictor of HE. Prior studies have indicated that the island sign, which may represent small arterial injury and bleeding adjacent to the hematoma, is a reliable imaging marker for predicting HE (Morotti et al., 2020). This study revealed smoking history, island sign, and maximum distance of the PHE as independent predictors for constructing the clinical model, which attained AUCs of 0.761, 0.680, and 0.660 in the training, testing, and validation sets, respectively.

Prior studies have established PHE as a quantifiable biomarker of brain injury and a potential therapeutic target in HICH (Cordonnier et al., 2018; Huang et al., 2023). PHE formation involves clot retraction, inflammation, and erythrolysis (Morotti et al., 2020), and accumulating evidence suggests that these processes are interrelated with HE (Florczak-Rzepka et al., 2012; Silva et al., 2004), and previous perfusion studies demonstrating reduced cerebral blood flow of the perihematomal region (Morotti et al., 2019; Morotti et al., 2020), implicating the perihematomal region in the pathophysiology of HE. In the present study, the maximum distance of the PHE was significantly greater in the HE group than in the NHE group across the training, testing, and validation sets, and it emerged as an independent predictor of HE. Motivated by this finding, we performed an in-depth analysis of PHE by applying a three-dimensional automated dilation technique to generate three concentric shells (5 mm, 10 mm, and 15 mm) around the manually delineated hematoma, from which radiomics features were separately extracted. The PHE 10 mm_radscore demonstrated the best predictive performance for HE, achieving AUCs of 0.839 (95% CI: 0.775–0.904) in the training set, 0.868 (95% CI: 0.772–0.963) in the testing set, and 0.834 (95% CI: 0.759–0.909) in the validation set. These results compare favorably with those of a previous radiomics study based on manual PHE delineation, which reported AUCs of 0.808 in the training cohort and 0.739 in the validation cohort (Zhou et al., 2024). Nevertheless, the optimal distance for PHE-based prediction of HE remains an unsettled question. A recent study reported that an “intrahematomal + automated perihematomal 14 mm” model yielded the highest AUC (0.899) in the test set (Wang et al., 2026); however, that study did not individually compare the performance of each automated perihematomal region. Besides, all cases in our cohort were deep HICH, the 5–15 mm dilation range largely avoids the skull, and we therefore did not apply a whole-brain mask to constrain the automated PHE expansion. Although this approach may allow partial inclusion of the ventricular and cerebrospinal fluid, such information could potentially carry additional predictive value (Wang et al., 2026). Moreover, this simplified strategy aligns with a rapid workflow and obviates the need for additional brain segmentation, making it more suitable for clinical practice.

The diagnosis of HICH relies substantially on NCCT. It has been demonstrated that numerous clinical feature and traditional CT imaging indicators constitute separate risk factors for HE (Hu et al., 2021; Li et al., 2019; Liu et al., 2019). Furthermore, radiomics, as an emerging technology, provides new methods for predicting HE (Tenhoeve et al., 2023; Chen et al., 2021). Although habitat analysis is predominantly applied in oncology research, its core of quantifying intralesional heterogeneity to reveal its underlying biological behavior has broad applicability. Following HICH, the formation of the hematoma is a dynamic evolving process involving the transformation from a suspension of red blood cells into a clot (Li et al., 2021; Powers et al., 2015; Magid-Bernstein et al., 2022). The co-existence of these different components provides the pathological basis for habitat analysis and radiomics to extract the heterogeneity of the hematoma. Conventional radiomics derives characteristics from the entire hematoma, however, as it cannot capture spatial distribution information, it struggles to achieve accurate clustering of similar voxels. In contrast, habitat analysis can identify and quantify the spatial distribution information of different pathological components within the hematoma, offering a more intuitive representation of the heterogeneity and complexity both within and between subregions. Previous study (Yu et al., 2023) has demonstrated that high intra-hematoma heterogeneity may reflect hematoma instability and could be associated with ongoing bleeding. By capturing this heterogeneity information, which corresponds to the underlying pathological mechanisms, habitat analysis exhibits a closer association with HE. The habitat radiomics model in our study showed a higher AUC for HE risk prediction in the validation set than the whole hematoma radiomics model. This indicates that spatially resolving the internal heterogeneity of the hematoma can provide predictive value beyond that of whole hematoma analysis, offering a further imaging perspective for understanding the mechanisms of HE.

Recent studies have shown that early HE can be predicted using radiomics and clinical data. Yu et al. (2023) developed hybrid models integrating radiomics features with clinical data, achieving AUC values of 0.901 in the training set, 0.838 in the validation set, and 0.917 in the testing set. Ye et al. (2023) developed ML models combining Rad-score with clinical indicators, with the best model achieving AUCs of 0.89 and 0.85 in the training and validation datasets, respectively. In comparison, our combined model—which integrates clinical features, conventional imaging features with whole hematoma, PHE, and habitat radiomics features—yielded consistently higher AUCs of 0.951, 0.937, and 0.939 in the training, testing, and validation sets, representing an improvement over the prior benchmarks across all cohorts. This performance gain may be attributable to our multi regional strategy, as incorporating PHE and habitat features captures complementary information from the perihematomal microenvironment that prior single region or dual region approaches may have overlooked.

This research possesses multiple limitations. First, as a retrospective study, it is naturally susceptible to selection bias. Although data were independently collected from two institutions, the relatively modest sample size and the inclusion of only two centers represent additional limitations. Larger prospective multicenter investigations are required for further validation. Second, the lack of some key clinical information, including the Glasgow coma scale and National institutes of health stroke scale scores at admission, as well as anticoagulant/antiplatelet use, due to its retrospective design. Although the absence of these clinical details may introduce confounding, our imaging-based biomarkers are designed to capture underlying biology. Nevertheless, prospective validation remains a necessary next step. Third, our study cohort consisted exclusively of Han Chinese patients, which may limit the generalizability of our findings to other racial and ethnic populations. Fourth, manual lesion segmentation may result in observer bias. To address this, two radiologists separately defined the ROIs, and interobserver agreement was evaluated using ICC. Nevertheless, the development of robust automated segmentation methods tailored to these complex boundaries remains an important goal to reduce variability and enhance reproducibility in future work. Fifth, considerable controversy remains regarding the optimal distance for PHE-based prediction of HE and the optimal timing of PHE measurement, both of which represent key scientific questions that merit further investigation in future studies.

The combined model established by clinical data with whole hematoma radiomics, habitat radiomics of the hematoma, and PHE radiomics features based on NCCT enhances HE prediction in patients with HICH. This noninvasive approach to HE prediction may inform therapeutic strategies and guide clinical management in HICH patients.

## Data Availability

The original contributions presented in the study are included in the article/Supplementary material, further inquiries can be directed to the corresponding authors.

## Footnotes

1
http://www.nitrc.org/projects/itk-snap/

2
www.r-project.org
