# Supplementary material for: Enhancing hematoma expansion prediction in hypertensive intracerebral hemorrhage based on habitat and perihematomal edema radiomics from non-contrast CT: a dual-center study
Source: Front Neurosci. 2026 Jun 24;20:1825573. doi: 10.3389/fnins.2026.1825573 (PMC13342042; doi:10.3389/fnins.2026.1825573)
Supplement: Supplementary file 1 [file Data_Sheet_1.PDF]

Radiomics features:

(1) shape features:

shape_Elongation
shape_Flatness
shape_LeastAxisLength
shape_MajorAxisLength
shape_Maximum2DDiameterColumn
shape_Maximum2DDiameterRow
shape_Maximum2DDiameterSlice
shape_Maximum3DDiameter
shape_MeshVolume
shape_MinorAxisLength
shape_Sphericity
shape_SurfaceArea
shape_SurfaceVolumeRatio
shape_VoxelVolume

(2) first-order features:

firstorder_10Percentile
firstorder_90Percentile
firstorder_Energy
firstorder_Entropy
firstorder_InterquartileRange
firstorder_Kurtosis
firstorder_Maximum
firstorder_MeanAbsoluteDeviation
firstorder_Mean
firstorder_Median
firstorder_Minimum
firstorder_Range
firstorder_RobustMeanAbsoluteDeviation
firstorder_RootMeanSquared
firstorder_Skewness
firstorder_TotalEnergy
firstorder_Uniformity
firstorder_Variance

(3) texture features:

glcm_Autocorrelation
glcm_ClusterProminence
glcm_ClusterShade
glcm_ClusterTendency
glcm_Contrast
glcm_Correlation
glcm_DifferenceAverage
glcm_DifferenceEntropy

glcm_DifferenceVariance
glcm_Id
glcm_Idm
glcm_Idmn
glcm_Idn
glcm_Imc1
glcm_Imc2
glcm_InverseVariance
glcm_JointAverage
glcm_JointEnergy
glcm_JointEntropy
glcm_MCC
glcm_MaximumProbability
glcm_SumAverage
glcm_SumEntropy
glcm_SumSquares
glrlm_GrayLevelNonUniformity
glrlm_GrayLevelNonUniformityNormalized
glrlm_GrayLevelVariance
glrlm_HighGrayLevelRunEmphasis
glrlm_LongRunEmphasis
glrlm_LongRunHighGray
glrlm_LongRunLowGrayLevelEmphasis
glrlm_LowGrayLevelRunEmphasis
glrlm_RunEntropy
glrlm_RunLengthNonUniformity
glrlm_RunLengthNonUniformityNormalized
glrlm_RunPercentage
glrlm_RunVariance
glrlm_ShortRunEmphasis
glrlm_ShortRunHighGrayLevelEmphasis
glrlm_ShortRunLowGrayLevelEmphasis
glszm_GrayLevelNonUniformity
glszm_GrayLevelNonUniformityNormalized
glszm_GrayLevelVariance
glszm_HighGrayLevelZoneVariance
glszm_LargeAreaEmphasis
glszm_LargeAreaHighGrayLevelEmphasis
glszm_LargeAreaLowGrayLevelEmphasis
glszm_LowGrayLevelZoneEmphasis
glszm_SizeZoneNonUniformity
glszm_SizeZoneNonUniformityNormalized
glszm_SmallAreaEmphasis
glszm_SmallAreaHighGrayLevelEmphasis

glszm_SmallAreaLowGrayLevelEmphasis
glszm_ZoneEntropy
glszm_ZonePercentage
glszm_ZoneVariance
gldm_DependenceEntropy
gldm_DependenceNonUniformity
gldm_DependenceNonUniformity Normalized
gldm_DependenceVariane
gldm_GrayLevelNonUniformity
gldm_GrayLevelVariance
gldm_HighGrayLevelEmphasis
gldm_LargeDependenceEmphasis
gldm_LargeDependenceHighGrayLevelEmphasis
gldm_LargeDependenceLowGrayLevelEmphasis
gldm_LowGrayLevelEmphasis
gldm_SmallDependenceEmphasis
gldm_SmallDependenceHighGrayLevelEmphasis
gldm_SmallDependenceLowGrayLevelEmphasis
ngtdm_Busyness
ngtdm_Coarseness
ngtdm_Complexity
ngtdm_Contrast
ngtdm_Strength

Supplementary Table S1 Prediction Performance of PHE radscore for Early Hematoma Expansion in Hypertensive Intracerebral Hemorrhage

		AUC (95% CI)	SEN	SPE	ACC	PPV	NPV
Training set	PHE 5mm_radscore	0.848 (0.787-0.908)	0.852	0.760	0.791	0.648	0.908
	PHE 10mm_radscore	0.839 (0.775-0.904)	0.852	0.702	0.753	0.597	0.901
	PHE 15mm_radscore	0.876 (0.824-0.928)	0.963	0.664	0.766	0.598	0.972
Testing set	PHE 5mm_radscore	0.844 (0.737-0.950)	0.826	0.733	0.765	0.613	0.892
	PHE 10mm_radscore	0.867 (0.772-0.963)	0.870	0.756	0.794	0.645	0.919
	PHE 15mm_radscore	0.767 (0.648-0.886)	0.870	0.556	0.662	0.500	0.893
Validation set	PHE 5mm_radscore	0.747 (0.663-0.831)	0.047	0.988	0.669	0.667	0.669
	PHE 10mm_radscore	0.834 (0.663-0.831)	0.814	0.726	0.756	0.603	0.884
	PHE 15mm_radscore	0.620 (0.517-0.722)	0.372	0.821	0.669	0.516	0.719

AUC:area under the curve; SEN: sensitivity; SPE: specificity; ACC: accuracy; PPV: positive predictive value; NPV: negative predictive value; PHE: perihematomal edema.

Supplementary Table S2. Confusion matrices of the combined model across all cohorts.

Cohort		Predicted HE	Predicted NHE	Total
Training	Actual HE	50	4	54
	Actual NHE	11	93	104
Testing	Actual HE	19	4	23
	Actual NHE	7	38	45
Validation	Actual HE	36	7	43
	Actual NHE	6	78	84

HE, hematoma expansion; NHE, non-hematoma expansion; TP, true positive; FN, false negative; FP, false positive; TN, true negative.

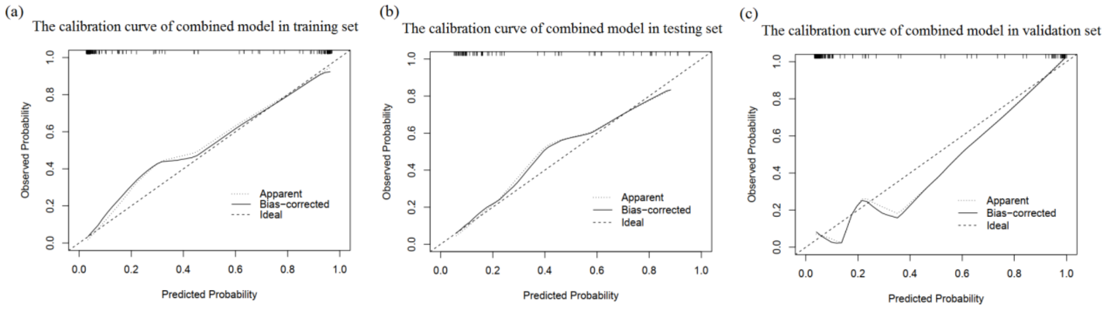

Supplementary Fig. S1 The calibration curves of the combined model in training set, testing set, and validation set.
